# Assessing retina-specific ophthalmic counseling generated by an early public large language model across different levels of clinical urgency

**DOI:** 10.3389/fdgth.2026.1849883

**Published:** 2026-07-01

**Authors:** Dominic M. Choo, Tyler A. Durham, Kishan G. Patel

**Affiliations:** Department of Ophthalmology, University of Texas Southwestern Medical Center, Dallas, TX, United States

**Keywords:** ChatGPT, clinical urgency, empathy, large language model, medical counseling, ophthalmology, readability, retina

## Abstract

**Purpose:**

To evaluate how the quality of retina-specific ophthalmology counseling provided by an early publicly available large language model (LLM) differs when advising patients with varying clinical characteristics and risk factors.

**Design:**

Prospective, cross-sectional study.

**Subjects:**

18 ophthalmologists.

**Methods:**

Six patient vignettes were constructed with high- and low-urgency clinical scenarios for diabetic retinopathy (DR), retinal detachment (RD), and age-related macular degeneration (AMD). Based on these vignettes, an LLM (ChatGPT-3.5) was asked to provide written medical counseling in February 2024. Each response was rated on several metrics via 5-point Likert scale by 18 independent reviewers. Notably, readability was assessed both qualitatively via survey and quantitatively via Readable (an online readability tool that incorporates 5 different metrics).

**Main outcome measures:**

Counseling generated by the LLM was graded on accuracy, appropriate communication of urgency and empathy, readability, and potential for clinically significant harm.

**Results:**

Counseling accuracy differed across levels of clinical urgency (*p* = 0.002) but remained consistent between high- and low-urgency vignettes of AMD (*p* = 0.081) and DR (*p* = 0.5), albeit not for RD (*P* < 0.001). Counseling urgency did not differ significantly from clinical urgency of all vignettes, except for the high-urgency AMD (*p* = 0.013) and high-urgency RD (*p* < 0.001). While counseling urgency did not significantly differ between high- and low-urgency vignettes of AMD (*p* = 0.055) and RD (*p* = 0.3), it did differ for the DR vignettes (*p* < 0.001). Counseling empathy did not differ across clinical urgency (*p* = 0.2). Four readability indices (e.g., Flesch Kincaid Grade Level) consistently indicated that college graduation would be required to understand every counseling output. Across all vignettes, the most common reasons for potential difficulty in understanding the counseling were having too much medical (49%) and non-medical (45%) terminology.

**Conclusions:**

The evaluated LLM-generated counseling outputs were largely similar across the sampled retinal vignettes with differing clinical urgency. Future studies should investigate the optimization of LLM prompting needed to garner counseling of consistent/appropriate accuracy, readability, empathy, and communication of urgency for specific conditions.

## 1 Introduction

Large Language Model (LLM) chatbots have become an increasingly popular source of information due to their unprecedented ability to distill and present data. For example, since the release of ChatGPT in November 2022, the program has accrued over 300 million weekly active users worldwide (1). Given how often patients use the internet for health-related queries, it is imperative that the capabilities of LLMs in this context are carefully evaluated (2).

Previous ophthalmology studies have demonstrated encouraging results. In one study, LLMs achieved an accuracy between 42.7%–59.4% on simulated Ophthalmic Knowledge Assessment Program questions (3). In another, LLMs tasked with patient case management largely matched or outperformed human glaucoma and retina subspecialists (4). Notably, the accuracy and readability of health-related information has been shown to vary across specialty and diseases (3, 4). However, the specific clinical characteristics of patients that may influence the quality of medical counseling have yet to be well characterized. This study aimed to assess the quality of ophthalmic counseling generated by an early publicly available LLM across different levels of clinical urgency.

## 2 Methods

### 2.1 Clinical vignette creation

Six clinical vignettes with retinal pathology were created ad-lib from the experience of a single investigator (K.G.P.) - a board-certified, retina subspecialist (Supplementary Data Sheet 1). The vignettes were intentionally designed to represent a dichotomy of clinical urgency, contrasting high- and low-urgency clinical scenarios for diabetic retinopathy (DR), retinal detachment (RD), and age-related macular degeneration (AMD) while maintaining similar levels of detail. To improve consistency across cases, each vignette was standardized with respect to overall length, sentence structure, degree of clinical detail influencing urgency and prognosis, (e.g., visual acuity, chronicity, disease severity, follow-up reliability. Specifically, within each disease category, the paired scenarios were constructed to differ primarily in perceived clinical urgency while maintaining similar informational complexity and readability. The vignettes were reviewed by the study team for clarity, realism, and consistency with typical retina clinic counseling scenarios prior to use.

### 2.2 LLM chatbot prompting

Vignettes were provided to an LLM (ChatGPT-3.5) by a single investigator (T.A.D.) in February 2024 in a brand new account. At the time, ChatGPT-3.5 was the most widely used version of ChatGPT as it was the only iteration available freely to the public. Via prompting, authors defined its role as an ophthalmologist, delivering concise medical counseling regarding the current state of the patient's eye disease, what caused their disease, and treatment recommendations (Supplementary Data Sheet 2). After this initial prompting, each clinical vignette (Supplementary Data Sheet 1) was inputted into the LLM. To minimize contextual carryover between cases, each vignette was entered into a newly initiated chat session with no prior conversation history or retained contextual memory. Each clinical vignette received one response from the LLM. The complete LLM-generated responses can be found in Supplementary Data Sheet 3.

### 2.3 Study design and participants

This prospective, cross-sectional, survey study was approved by The University of Texas Southwestern Medical Center Institutional Review Board and followed all tenets of the Declaration of Helsinki. A cohort of ophthalmologists with varying degrees of fellowship experience were asked to rate the LLM-generated medical counseling via an online survey.

The survey included a set of six questions (five Likert scale, one choose-all-that-apply) for each vignette (Supplementary Data Sheet 3). All Likert scale questions were asked on a 5-point scale where only the extremes were qualitatively defined (e.g., 1 = “very low risk of harm” vs. 5 = “very high risk of harm”). Furthermore, each survey was ordered so that analogous/paired vignettes were separated.

All surveys were distributed via Microsoft Forms on July 1, 2024 and completed independently within 3 weeks. Participants rated the LLM-generated responses in a masked fashion (i.e., they did not know it was generated by artificial intelligence). Ultimately, this resulted in a total of 648 ratings across several variables. Separate from the survey, researchers additionally scored counseling readability using objective metrics.

### 2.4 Objectives and endpoints

Vignettes were rated for clinical urgency. LLM-generated counseling was rated on the following metrics via Likert scale: accuracy, amount of conveyed urgency, potential for clinically significant harm, amount of empathy, appropriateness of empathy. Readability was qualitatively assessed via a choose-all-that-apply question regarding potential barriers to readability, and quantitatively assessed using 4 indices - the Flesch Kincaid Grade Level, Gunning Fog Index, Simple Measure of Gobbledygook (SMOG) Index, Coleman-Liau Index and the New Dale-Chall Readability Formula (1995) - via the online readability application, Readable (5). Indices were chosen following literature precedent (6–8).

### 2.5 Statistical analysis

Ordinal categorical data are presented as counts with percentages. While Likert scales are traditionally treated as ordinal, medians with interquartile ranges were also presented, treating the Likert responses as interval data. The labeling of the Likert scale from 1 to 5 with only extremes qualitatively defined suggests to respondents that there is an equal distance between each number in the scale. Due to the nature of Likert scale data and the non-normal distribution of the data, the Kruskal–Wallis test (a nonparametric test) was used to descriptively compare rating distributions across vignette groups. Note that since the test does not fully account for within-rater correlation stemming from the repeated-measures structure, rendering the analyses exploratory and descriptive. The level of significance was set at *p* <0.05. Statistics, tables, and figures were generated using R software (version 4.4.2, R Core Team) using the following packages: “gtsummary”, “irr”, “psych” (9–11). Data available upon request.

A primary analysis was done to compare counseling between vignettes of high- and low-urgencies within each retinal disease. Subsequently, a secondary analysis was conducted to compare counseling across vignette “urgency scores”, ignoring retinal pathology. To do so, urgency scores were assigned to each vignette based on the median physician rating of their urgency (i.e., “What degree of urgency would you, personally, counsel the patient with?”).

A sensitivity analysis was also performed to investigate whether participants rated the quality of counseling independently from their interpretation of the vignette's clinical urgency (Supplementary Data Sheet 4). Instead of stratifying participants' ratings of counseling quality by median vignette clinical urgency scores, they were stratified by each participant's individual rating of each vignette's clinical urgency. Finally, a second sensitivity analysis was done in which the primary and secondary analyses (Supplementary Data Sheet 5, 6, respectively) were repeated using only responses from retinal subspecialists (defined as board-certified attending ophthalmologists who had previously completed vitreoretinal disease and surgery fellowship).

## 3 Results

### 3.1 Participant demographics

This study had 18 participants - including 11 board-certified and fellowship-trained retina subspecialists, 1 board-certified and fellowship-trained uveitis specialist, 2 board-certified general ophthalmologist, and 4 current vitreoretinal surgery fellows.

### 3.2 Survey question and response reliability

To assess rater consistency for the Likert scale questions, we calculated the Intraclass Correlation Coefficient (ICC) using a two-way mixed effects model for average measures and obtained a value of 0.94, indicating excellent agreement (12). The ICCs for each survey question/grading metric can be found in Supplementary Data Sheet 7. For the subsequent choose-all-that-apply question, Fleiss' Kappa was calculated and yielded a value of 0.103, suggesting only slight agreement among raters (Supplementary Data Sheet 7) (13). Survey responses were not normally distributed.

Physician ratings of vignette urgency corroborated the authors' intended differences in clinical urgency between the high- and low-urgency vignettes within each retinal pathology (Table 1). All cases intended by the authors to be high-urgency received a median urgency rating between 4 and 5 (1 = “very low urgency” vs. 5 = “very high urgency”), while all cases intended to be low-urgency had a median rating of 3. All vignettes were given a median vignette score of 3–5; no vignettes were rated with an urgency of 1 or 2.

**Table 1:** Descriptive statistics of survey responses by disease by risk level.

	Age-Related Macular Degeneration			Diabetic Retinopathy			Retinal Detachment		
Metric	High-Risk *N* = 18^a^	Low-Risk *N* = 18^a^	*p*-value^b^	High-Risk *N* = 18^a^	Low-Risk *N* = 18^a^	*p*-value^b^	High-Risk *N* = 18^a^	Low-Risk *N* = 18^a^	*p*-value^b^
Counseling accuracy	5.00 (4.00, 5.00)	4.00 (3.00, 5.00)	0.081	5.00 (4.00, 5.00)	4.50 (4.00, 5.00)	0.5	5.00 (4.00, 5.00)	3.00 (3.00, 4.00)	<0.001
Vignette's urgency	4.00 (4.00, 4.00)	3.00 (2.00, 4.00)	<0.001	4.00 (4.00, 5.00)	3.00 (2.00, 3.00)	<0.001	5.00 (5.00, 5.00)	3.00 (2.00, 4.00)	<0.001
Counseling urgency	4.00 (3.00, 4.00)	3.00 (3.00, 4.00)	0.055	4.00 (3.00, 4.00)	3.00 (2.00, 3.00)	<0.001	4.00 (3.00, 4.00)	3.50 (3.00, 4.00)	0.3
Clinically significant harm	1.50 (1.00, 2.00)	2.00 (1.00, 2.00)	0.3	1.50 (1.00, 3.00)	1.00 (1.00, 2.00)	0.4	2.00 (1.00, 3.00)	2.00 (2.00, 3.00)	0.2
Counseling empathy	4.00 (3.00, 4.00)	3.00 (2.00, 3.00)	0.007	3.00 (2.00, 3.00)	3.00 (3.00, 4.00)	0.15	3.50 (3.00, 4.00)	3.00 (2.00, 3.00)	0.072
Appropriateness of counseling empathy	4.00 (3.00, 5.00)	3.00 (3.00, 4.00)	0.019	3.00 (2.00, 4.00)	4.00 (3.00, 5.00)	0.092	3.50 (3.00, 5.00)	3.00 (2.00, 4.00)	0.2
Understandability to average, native English-speaking patient	4.00 (3.00, 5.00)	3.00 (2.00, 5.00)	0.063	3.00 (2.00, 4.00)	3.50 (3.00, 4.00)	0.13	3.00 (2.00, 4.00)	3.00 (2.00, 3.00)	0.3
Little to no difficulties^c^	7 (39%)	4 (22%)	0.3	4 (22%)	6 (33%)	0.5	2 (11%)	3 (17%)	>0.9
Too much medical terminology^c^	5 (28%)	11 (61%)	0.044	10 (56%)	8 (44%)	0.5	10 (56%)	9 (50%)	0.7
Difficult non-medical word choice^c^	6 (33%)	10 (56%)	0.2	11 (61%)	7 (39%)	0.2	6 (33%)	9 (50%)	0.3
Lack of semantic organization^c^	0 (0%)	2 (11%)	0.5	1 (5.6%)	0 (0%)	>0.9	2 (11%)	5 (28%)	0.4
Inadequate information^c^	4 (22%)	5 (28%)	>0.9	0 (0%)	1 (5.6%)	>0.9	4 (22%)	6 (33%)	0.5
Too much unnecessary information^c^	4 (22%)	3 (17%)	>0.9	10 (56%)	7 (39%)	0.3	7 (39%)	7 (39%)	>0.9

a Median (Q1, Q3); *n* (%).

b Kruskal–Wallis rank sum test; Pearson's Chi-squared test; Fisher's exact test.

c Presence of readability issue.

Furthermore, metrics of grading counseling did not differ significantly when solely stratified by retinal pathology (i.e., AMD, DR, RD), except for vignette urgency (*p* = 0.044), potential for clinically significant harm (*p* = 0.047), and “readability issue: inadequate information” (*p* = 0.011) (Supplementary Data Sheet 8).

### 3.3 Accuracy

Overall, counseling accuracy was rated highly across all vignettes with medians of 4–5 (1 = “very low accuracy” vs. 5 = “very high accuracy”), except for low-urgency RD (median of 3; Table 1). Counseling accuracy differed significantly across vignette urgency scores (*p* = 0.002; Table 2. Moreover, when compared between high- and low-urgency within each retinal pathology, accuracy did not differ significantly for AMD (*p* = 0.081) and DR (*p* = 0.5) but not for RD (*p* < 0.001; Table 1). Of all counseling responses, the high-urgency RD counseling received the highest accuracy ratings [4 (33%), 5 (67%)]; in contrast, the low-urgency RD case received the lowest distribution of ratings [2(22%), 3(33%), 4(22%), 5(22%)].

**Table 2:** Descriptive statistics of survey responses by urgency score.

	Urgency Score (Median Physician Rating of Vignette's Urgency)			
Metric	3 *N* = 54^a^	4 *N* = 36^a^	5 *N* = 18^a^	*p*-value^b^
Counseling accuracy	4.00 (3.00, 5.00)	5.00 (4.00, 5.00)	5.00 (4.00, 5.00)	0.002
Counseling urgency	3.00 (3.00, 4.00)	4.00 (3.00, 4.00)	4.00 (3.00, 4.00)	<0.001
Clinically significant harm	2.00 (1.00, 3.00)	1.50 (1.00, 2.00)	2.00 (1.00, 3.00)	0.5
Counseling empathy	3.00 (2.00, 4.00)	3.00 (3.00, 4.00)	3.50 (3.00, 4.00)	0.2
Appropriateness of counseling empathy	3.00 (3.00, 4.00)	4.00 (3.00, 4.50)	3.50 (3.00, 5.00)	0.6
Understandability to average, native English-speaking patient	3.00 (2.00, 4.00)	3.00 (2.50, 4.00)	3.00 (2.00, 4.00)	0.6
Little to no difficulties^c^	13 (24%)	11 (31%)	2 (11%)	0.3
Too much medical terminology^c^	28 (52%)	15 (42%)	10 (56%)	0.5
Difficult non-medical word choice^c^	26 (48%)	17 (47%)	6 (33%)	0.5
Lack of semantic organization^c^	7 (13%)	1 (2.8%)	2 (11%)	0.2
Inadequate information^c^	12 (22%)	4 (11%)	4 (22%)	0.4
Too much unnecessary information^c^	17 (31%)	14 (39%)	7 (39%)	0.7

a Median (Q1, Q3); *n* (%).

b Kruskal–Wallis rank sum test; Fisher's exact test; Pearson's Chi-squared test.

c Presence of readability issue.

### 3.4 Appropriate communication of clinical urgency

The amount of urgency conveyed in counseling showed statistically significant differences across vignette urgency scores (*p* < 0.001; Table 2). In doing so, counseling urgency had a positive trending relationship with vignette urgency (Table 2). When clinical vignettes were grouped by their median urgency score, and the distribution of urgency scores for these categories were compared to the distribution of counseling urgency ratings, a statistically significant difference was observed at all urgency scores [3 [*p* = 0.040], 4 [*p* = 0.007], 6 [*p* < 0.001]; Table 3]. When stratified by retinal pathologies, counseling urgency did not show statistical differences between the author-intended high- and low-urgency AMD (*p* = 0.055) and RD (*p* = 0.3) but did for DR (*p* < 0.001; Table 1). When all vignettes were analyzed individually, counseling urgency did not differ significantly from each vignette's urgency score except for high-urgency AMD (*p* = 0.013) and high-urgency RD (*p* < 0.001; Table 4).

**Table 3:** Comparison of counseling urgency and vignette urgency across urgency scores.

Urgency Score^a^	Rating of the vignette urgency *N* = 54^b^	Rating of counseling urgency *N* = 54^b^	*p*-value^c^
3	3.00 (2.00, 3.00)	3.00 (3.00, 4.00)	0.040
4	4.00 (4.00, 5.00)	4.00 (3.00, 4.00)	0.007
5	5.00 (5.00, 5.00)	4.00 (3.00, 4.00)	<0.001

a Urgency score, median physician rating of vignette urgency

b Median (Q1, Q2); *n* (%).

c Kruskal–Wallis Rank sum test.

**Table 4:** Comparison of counseling urgency and vignette urgency across all vignettes.

Retinal Pathology	Author Assigned Urgency	Rating of the vignette urgency *N* = 18^a^	Rating of counseling urgency *N* = 18^a^	*p*-value^b^
AMD	High	4.00 (4.00, 4.00)	4.00 (3.00, 4.00)	0.013
	Low	3.00 (2.00, 4.00)	3.00 (3.00, 4.00)	0.5
DR	High	4.00 (4.00, 5.00)	4.00 (3.00, 4.00)	0.2
	Low	3.00 (2.00, 3.00)	3.00 (2.00, 3.00)	0.4
RD	High	5.00 (5.00, 5.00)	4.00 (3.00, 4.00)	<0.001
	Low	3.00 (2.00, 4.00)	3.50 (3.00, 4.00)	0.053

a Median (Q1, Q3); *n* (%).

b Kruskal–Wallis rank sum test.

### 3.5 Appropriate communication of empathy

Overall, counseling empathy was rated neither highly nor lowly, with all counseling responses being rated with a median of 3 except for high-urgency AMD (4) and high-urgency RD (3.5; Table 1). Across clinical urgency scores, neither the amount of empathy conveyed, nor the appropriateness of conveyed empathy significantly differed (*p* = 0.2, *p* = 0.6, respectively; Table 2). When stratified by retinal pathology, counseling empathy differed between high- and low-urgency AMD (*p* = 0.007) but not for DR (*p* = 0.15) and RD (*p* = 0.072; Table 1); ratings for appropriateness of empathy conveyed only differed significantly for AMD (*p* = 0.019).

### 3.6 Potential for clinically significant harm

Overall, the potential risk for clinically significant harm arising from the LLM-generated counseling was rated low across all vignettes ranging from 1 to 2 (1 = “very low risk of harm” vs. 5 = “very high risk of harm”). Within each retinal pathology, risk did not differ significantly between high- and low-urgency vignettes [AMD (*p* = 0.3), DR (*p* = 0.4), RD (*p* = 0.2); Table 1]. This also held true across vignette clinical urgency scores (*p* = 0.5; Table 2).

### 3.7 Readability

When asked “How well do you feel that your average, native English-speaking patient would understand the response” with 1 being “little to no understanding” and 5 being “excellent understanding”, physicians gave all vignettes a median score of 3, except for high-urgency RD (4) and low-urgency DR (3.5; Table 1). Readability ratings did not differ significantly across clinical urgency score (*p* = 0.6; Table 2), as well as between high- and low-urgency vignettes within each retinal pathology [AMD (*p* = 0.063), DR (*p* = 0.13), RD (*p* = 0.3); Table 1].

When asked to denote what kinds of issues with readability the same patients were likely to experience, physicians marked “too much medical terminology” and “difficult non-medical word choice” most frequently across all vignettes (49% and 45%, respectively). This remained the same when cases were grouped by retinal pathology [AMD (44%, 44%), DR (50%, 50%), RD (53%, 42%), respectively]. Within each retinal pathology, the high- and low-urgency cases did not show significant differences except for AMD (*p* = 0.044) - “too much medical terminology” was marked by 21% and 61%, respectively. None of the readability issue options were marked with significant differences in frequency across urgency scores (Table 2). Additionally, the objective readability scores in Table 5 indicated all counseling to be difficult or very difficult to read for the average lay person—college graduation would be required to understand the material.

**Table 5:** Objective readability metrics.

Retinal Pathology	Author Assigned Urgency	Median Clinical Urgency Score	FKG	GFI	CLI	SMOG Index	New Dale-Chall
DR	High	4	15.7	18.3	15.6	16.7	8.6
	Low	3	15.2	17.8	16.8	16.3	16.2
AMD	High	4	14.8	18.1	16.1	16.2	11.5
	Low	3	16.8	20.4	17.1	18.0	7.7
RD	High	5	14.3	18.6	15.1	16.4	12
	Low	3	15.2	20.0	16.0	17.5	16.1

DR, diabetic retinopathy; AMD, age-related macular degeneration; RD, retinal detachment; FKG, flesch-kincaid grade, GFI, gunning fog index; CLI, Coleman-Liau Index; SMOG, simple measure of gobbledygook.

### 3.8 Effect of participants' vignette urgency ratings on other counseling quality metrics

The sensitivity analysis performed to investigate whether participants rated the quality of counseling independently from their interpretation of the vignette's clinical urgency did not yield greater nor fewer statistically significant results from the secondary analysis (Table 2) which grouped vignettes clinical urgency scores.

### 3.9 Retina subspecialist data

The sensitivity analysis using only data from retina subspecialists (*n* = 11) corroborated nearly all previously revealed statistical differences. Overall, there were only 3 instances when the retina subspecialists demonstrated a statistically significant difference that the whole sample did not. When asked “How well do you feel that your average, native English-speaking patient would understand the response (5-point Likert scale)”, the retina subspecialists showed a statistical difference between high- and low-urgency AMD (*p* = 0.030; Supplementary Data Sheet 5), as well as high- and low-urgency RD (*p* = 0.042). Finally, they answered that the amount of empathy conveyed varied across clinical urgency scores (*p* = 0.037; Supplementary Data Sheet 6).

## 4 Discussion

Overall, the evaluated LLM-generated counseling was rated favorably on accuracy, appropriate usage of urgency, and low potential for causing clinically significant harm. On the other hand, counseling was rated on the lower end with empathy and readability. Accuracy was found to differ based on the vignette's clinical urgency; however, because each disease-urgency combination was represented by a single vignette, these differences may also reflect vignette-specific characteristics unrelated to urgency itself. The amount of urgency conveyed in counseling was largely found to be appropriate across vignette urgency scores; while the appropriateness of empathy was consistently rated on the lower end.

Previous studies demonstrated similar results when assessing the accuracy of LLM-generated medical information. Momenaei et al. (2023) found 85%–92% of ChatGPT-4.0 responses to questions related to RD, macular holes, and epiretinal membranes to be correct and similar to the recommendations of an ophthalmologist (6). A string of other studies demonstrated that LLM chatbots performed slightly behind or similar to ophthalmology residents and current attendings in various specialties (e.g., glaucoma, retina, uveitis) (4, 14, 15).

However, studies investigating ChatGPT's ability to stratify patients based on risk factors demonstrated less encouraging results (16–18). Zaboli et al. (2024) found ChatGPT to largely underperform triage nurses in stratifying emergency department patients during intake (16). Sievert et al. (2024) found ChatGPT to have an overall accuracy of 68% when distinguishing between low- and high-risk thyroid nodules (17). Another study by Heston and Lewis (2024) found ChatGPT-4 to have a high correlation with established risk stratification tools for acute nontraumatic chest pain (i.e., TIMI, HEART), but was too inconsistent when presented with identical patient data on separate occasions to be used for real patients (18).

Comparatively, the sampled ChatGPT-generated counseling outputs in this study showed similar patterns to physician-rated clinical urgency across several vignettes. Though urgency ratings differed when vignettes were grouped by their median urgency score, most counseling and vignette urgency scores did not differ significantly when vignettes were stratified by retinal pathology or individually. Notably, the potential for causing clinically significant risk was consistently rated on the lower end across all vignettes, and did not differ based on vignette clinical urgency scores across all analyses (i.e., primary, secondary, tertiary, quaternary). It should be noted, however, that the risk of clinically significant harm are based on subjective ratings rather than objective clinical validation. The amount of urgency conveyed in each counseling response did not differ significantly from the amount of urgency ophthalmologists said they would have used for the same patient for all vignettes except for high-urgency AMD and high-urgency RD. Interestingly, the LLM-generated responses had a difficult time modulating the amount of empathy conveyed based on the clinical urgency of the vignette; however, this may just be a general limitation to the chatbot as it displayed low amounts of empathy consistently across all vignettes.

Educational materials intended for patient use must meet specific requirements for readability. For example, the American Medical Association recommends a reading level between fifth and sixth grade for health-related information. The quantitative readability indices used in this study found the LLM-generated counseling to be too complex for the general population, which aligned with many previous studies (6–8). However, the ophthalmologists who participated in our study scored the readability either moderately or favorably. They found that around half of ChatGPT's responses to have too much medical jargon and difficult non-medical terminology; however, they only found issue with 10% and 20% of responses for semantic organization and completeness/cohesiveness of the response, respectively. Notably, the latter metrics are not included in the scoring system of the quantitative indices and may indicate ChatGPT's readability to be of more acceptable quality than previous studies may have implied and speak to the inadequacy of exclusively using the quantitative indices in assessing ChatGPT.

Our work has several limitations. First, this study evaluated a limited number of clinical vignettes representing only three retinal conditions, and therefore the findings may not generalize to other ophthalmic diseases or counseling scenarios and observed counseling differences may reflect vignette specific characteristics rather than urgency alone. Additionally, though the cases were designed to represent varying levels of clinical urgency, the vignette urgency scores demonstrated a relatively narrow median range between 3 and 5, limiting representation of the full spectrum of ophthalmic urgency. Second, response appropriateness was assessed using subjective physician grading. Although the intraclass correlation coefficient demonstrated high inter-rater reliability for overall grading, our reviewers were obtained through convenience sampling, limiting the generalizability of these findings to the broader ophthalmology community. The relatively low Fleiss' kappa for readability-related issues also suggests limited agreement among reviewers in this domain, weakening conclusions related to readability assessments. Including readability assessments based on patient perspectives could have also provided additional insight beyond physician-based evaluation. Several statistical limitations should also be considered. Median values were used to assign urgency scores because survey responses were non-normally distributed; however, this approach reduced discrimination between vignettes of similar urgency compared with continuous measures of central tendency. Additionally, Likert scale responses were analyzed as interval variables, despite ordinal analysis generally being preferred for Likert data. An additional limitation is that the same reviewers evaluated all vignettes, creating a repeated-measures structure that may have introduced within-rater correlation not fully accounted for in the statistical analyses.

Finally, this study evaluated ChatGPT-3.5, an earlier publicly available large language model, and recorded one response per vignette. While newer and improved upon versions of ChatGPT and LLMs will continue to be released, this study provides a foundational assessment of AI-driven counseling within ophthalmology. The present study's evaluation of ChatGPT-3.5-generated counseling offers important insights into the baseline performance of AI in subspecialty-specific patient education, which will be critical for future and offers a replicable assessment of AI-driven counseling (19). Given the continuous evolution of LLMs, with each iteration presumably yielding superior results, the assessment of their performance in patient counseling will inherently lag behind the latest advancements. As these technologies advance at a rapid pace, traditional evaluation frameworks may struggle to keep up, necessitating iterable methodologies for ongoing assessment. Additionally, models developed by different companies have shown differences in performances as well. Recent comparative work across contemporary LLMs evaluating ChatGPT-4.0 Mini, Gemini 1.5 Flash, and Microsoft Copilot on ophthalmology examination questions demonstrated substantial variability in both accuracy and linguistic complexity. Given the novelty of this field, few studies have examined the effectiveness of LLMs in patient counseling. A detailed array of metrics and large number of ophthalmologist respondents relative to other comparable survey studies strengthens the present study.

Our results, although exploratory, suggest that the sampled ChatGPT-3.5-generated outputs evaluated in this study showed similar physician-rated patterns across different levels of clinical urgency, even when further stratified for retinal pathologies including diabetic retinopathy, retinal detachment, and macular degeneration. However, there is a need to assess the level of detail needed when prompting the LLM for medical counseling to ensure consistent levels of accuracy. The readability scores were also consistently low for the general audience. Further prompting with reading level requests, as well as the continuous development of AI-based technologies, may help in this regard.

## Data Availability

The raw data supporting the conclusions of this article will be made available by the authors, without undue reservation.
