# Supplementary material for: Assessing retina-specific ophthalmic counseling generated by an early public large language model across different levels of clinical urgency
Source: Front Digit Health. 2026 Jul 1;8:1849883. doi: 10.3389/fdgth.2026.1849883 (PMC13368933; doi:10.3389/fdgth.2026.1849883)
Supplement: Supplementary file 1 [file Datasheet1.pdf]

Supplement 1

Case 1
Patient is a 25 year old with Type 1 Diabetes Mellitus, Hemoglobin A1c 12%, and proliferative diabetic retinopathy, who has shown poor follow-up to clinic appointments.

Case 2
Patient is monocular, has peripheral retinal detachment in the monocular eye, and good visual acuity (20/20).

Case 3
Patient has 20/20 vision and new acute-onset wet macular degeneration.

Case 4
Patient is a 65 year old with Type 2 Diabetes Mellitus, Hemoglobin A1c 6.0%, and mild non-proliferative diabetic retinopathy, who has shown good follow-up to clinic appointments.

Case 5
Patient has an amblyopic eye, chronic total retinal detachment, and hand-motion vision.

Case 6
Patient has hand-motion vision and chronic wet macular degeneration.
