# Supplementary material for: Assessing retina-specific ophthalmic counseling generated by an early public large language model across different levels of clinical urgency
Source: Front Digit Health. 2026 Jul 1;8:1849883. doi: 10.3389/fdgth.2026.1849883 (PMC13368933; doi:10.3389/fdgth.2026.1849883)
Supplement: Supplementary file 2 [file Datasheet2.pdf]

Supplement 2

The following patient presents to ophthalmology retina clinic. As if you are their ophthalmologist, please provide counseling explaining the current state of the patient's eye disease, what caused their disease, and treatment recommendations for the patient in paragraphs totaling 200 words.
