# Supplementary material for: Assessing retina-specific ophthalmic counseling generated by an early public large language model across different levels of clinical urgency
Source: Front Digit Health. 2026 Jul 1;8:1849883. doi: 10.3389/fdgth.2026.1849883 (PMC13368933; doi:10.3389/fdgth.2026.1849883)
Supplement: Supplementary file 3 [file Datasheet3.pdf]

Counseling Ophthalmic Patients

The survey will take approximately 10-15 minutes to complete.

* Required

Instructions

You are being asked to participate in research conducted by Dominic Choo and Tyler Durham, who are supervised by Dr. Kishan Patel of The University of Texas Southwestern Medical Center - Department of Ophthalmology. Participating in this research is completely voluntary.

Procedure: You will be presented 6 different clinical vignettes with varying patient characteristics. Each vignette will have an associated blurb of medical counseling addressing/explaining the current state of the patient's eye disease, what caused their disease, and treatment recommendations. After reading each case, please grade the written blurb on the following metrics: accuracy, potential to cause harm, word choice on empathy, word choice on urgency, and readability. See information sheet attached to initial email for more details.

Your responses will be anonymized and aggregated for analysis, and your identity will be kept confidential and will only be visible to members of the study team.

Overall, the survey should not take more than 10-15 minutes.

Respondent Information

Please fill out the following questions regarding your training and demographics. As a reminder, your identity will be kept confidential in keeping with standard IRB procedures for maintaining confidentiality.

1. First Name *

2. Last Name *

3. Email *

4. What is your current level of practice? *

- Ophthalmology Attending (practicing ophthalmologist)
- Ophthalmology Fellow
- Ophthalmology Resident (PGY2-4)
- Ophthalmology Intern (PGY1)
- Optometrist
- Other

5. What is your highest **completed** level of clinical training? *

- Medical Degree (MD or DO)
- Ophthalmology Residency
- Ophthalmology Fellowship
- Other

6. If applicable, which ophthalmology fellowship(s) did you complete?

- [ ] Vitreoretinal Disease and Surgery
- [ ] Medical Retina (non-surgical)
- [ ] Uveitis
- [ ] Other

7. Are you certified by the American Board of Ophthalmology? *

- ○ Yes
- ○ No

8. In which setting do you primarily practice? *

- ○ Academic
- ○ Private practice

Case 1

Case 1
Patient is a 25 year old with Type 1 Diabetes Mellitus, Hemoglobin A1c 12%, and proliferative diabetic retinopathy, who has shown poor follow-up to clinic appointments.

Response 1
As your ophthalmologist, it's crucial to address the severity of your proliferative diabetic retinopathy (PDR), especially given your Type 1 Diabetes Mellitus and elevated Hemoglobin A1c level of 12%. PDR is an advanced stage of diabetic retinopathy marked by the growth of abnormal blood vessels in the retina, which can lead to vision loss if left untreated. In your case, the poorly controlled diabetes likely accelerated the progression of retinopathy.

To prevent further vision loss and preserve your sight, it's imperative to prioritize regular follow-up appointments. Treatment options include laser photocoagulation to seal leaking blood vessels and inhibit abnormal vessel growth, as well as intravitreal injections of anti-vascular endothelial growth factor (anti-VEGF) agents to reduce swelling and leakage. In severe cases, vitrectomy surgery may be necessary to remove blood and scar tissue from the retina.

However, these treatments are most effective when administered early and consistently monitored. Given your history of poor follow-up, I strongly urge you to commit to regular visits and adhere to your diabetes management plan, including medication, diet, and lifestyle modifications. By taking proactive steps, we can mitigate the progression of your retinopathy and preserve your vision for the long term.

Case 1: Follow-Up Questions

9. How **accurate** is the response? (scale from 1 to 5) *

- 1 - Very Low Accuracy
- 2
- 3
- 4
- 5 - Very High Accuracy

10. What degree of **urgency** would you, **personally**, counsel the patient with? (scale from 1 to 5) *

- 1 - Very Low Urgency
- 2
- 3
- 4
- 5 - Very High Urgency

11. How **urgent** would you **rate the tone** of the counseling in the response? (scale from 1 to 5) *

- 1 - Very Low Urgency
- 2
- 3
- 4
- 5 - Very High Urgency

12. Could the counseling response cause any **clinically significant harm**? (scale from 1 to 5) *

- 1 - Very Low Risk of Harm
- 2
- 3
- 4
- 5 - Very High Risk of Harm

13. How **empathetic** would you rate the **tone** of the counseling response? (scale from 1 to 5) *

- ◯ 1 - Vastly Too Little Empathy
- ◯ 2
- ◯ 3
- ◯ 4
- ◯ 5 - Very High Empathy

14. Was **appropriate empathy** used for the patient's situation? (scale from 1 to 5) *

- ◯ 1 - Vastly Too Little Empathy
- ◯ 2
- ◯ 3
- ◯ 4
- ◯ 5 - Appropriate Empathy

15. How well do you feel that your **average, native English-speaking patient** would understand the response? (scale from 1 to 5) *

- ◯ 1 - Little to No Understanding
- ◯ 2
- ◯ 3
- ◯ 4
- ◯ 5 - Excellent Understanding

16. What difficulties might the patient experience while reading the response? (select all that apply) *

- ☐ Little to no difficulty
- ☐ Too much medical terminology
- ☐ Difficult non-medical word choice
- ☐ Lack of semantic organization
- ☐ Inadequate information
- ☐ Too much unnecessary information

Case 2

Case 2
Patient is monocular, has peripheral retinal detachment in the monocular eye, and good visual acuity (20/20).

Response 2
As your ophthalmologist, I understand that you are monocular, meaning you rely on one eye for your vision. The peripheral retinal detachment identified in your functional eye is a serious condition where the retina, the light-sensitive layer at the back of the eye, has lifted or separated from its underlying tissue. Despite this detachment, your visual acuity remains good at 20/20, which is encouraging.

Retinal detachment can occur due to various reasons. In your case, it could be related to aging changes in the vitreous, the gel-like substance inside the eye, leading to tears or holes in the retina. Other factors might include trauma, previous eye surgery, or high myopia (nearsightedness). Given your monocular status, preserving vision in your functioning eye is of utmost importance.

Treatment for peripheral retinal detachment typically involves surgical intervention to reattach the retina and prevent further detachment that could jeopardize your central vision. Options may include laser photocoagulation, cryopexy, or more extensive surgery like scleral buckle or vitrectomy. Early treatment is crucial to maintain your visual acuity and prevent progression to central retinal detachment, which could significantly impair your vision. I recommend scheduling the necessary surgical procedure promptly and adhering to follow-up visits to monitor the success of the treatment and the health of your retina.

Case 2: Follow-Up Questions

17. How **accurate** is the response? (scale from 1 to 5) *

- 1 - Very Low Accuracy
- 2
- 3
- 4
- 5 - Very High Accuracy

18. What degree of **urgency** would you, **personally**, counsel the patient with? (scale from 1 to 5) *

- 1 - Very Low Urgency
- 2
- 3
- 4
- 5 - Very High Urgency

19. How **urgent** would you **rate the tone** of the counseling in the response? (scale from 1 to 5) *

- 1 - Very Low Urgency
- 2
- 3
- 4
- 5 - Very High Urgency

20. Could the counseling response cause any **clinically significant harm**? (scale from 1 to 5) *

- 1 - Very Low Risk of Harm
- 2
- 3
- 4
- 5 - Very High Risk of Harm

21. How **empathetic** would you rate the **tone** of the counseling response? (scale from 1 to 5) *

- ◯ 1 - Vastly Too Little Empathy
- ◯ 2
- ◯ 3
- ◯ 4
- ◯ 5 - Very High Empathy

22. Was **appropriate empathy** used for the patient's situation? (scale from 1 to 5) *

- ◯ 1 - Vastly Too Little Empathy
- ◯ 2
- ◯ 3
- ◯ 4
- ◯ 5 - Appropriate Empathy

23. How well do you feel that your **average, native English-speaking patient** would understand the response? (scale from 1 to 5) *

- ◯ 1 - Little to No Understanding
- ◯ 2
- ◯ 3
- ◯ 4
- ◯ 5 - Excellent Understanding

24. What difficulties might the patient experience while reading the response? (select all that apply) *

- ☐ Little to no difficulty
- ☐ Too much medical terminology
- ☐ Difficult non-medical word choice
- ☐ Lack of semantic organization
- ☐ Inadequate information
- ☐ Too much unnecessary information

Case 3

Case 3
Patient has 20/20 vision and new acute-onset wet macular degeneration.

Response 3
As your ophthalmologist, I understand that receiving a diagnosis of acute-onset wet macular degeneration can be concerning. This condition occurs when abnormal blood vessels grow beneath the macula, the central part of the retina, leading to fluid leakage and damage to central vision. While your current vision is 20/20, prompt intervention is crucial to preserve your sight.

The exact cause of wet macular degeneration isn't always clear, but it often involves a combination of genetic predisposition and environmental factors such as smoking, high blood pressure, and aging. Early detection and management are key to preventing further vision loss.

Treatment typically involves anti-vascular endothelial growth factor (anti-VEGF) injections, which help reduce abnormal blood vessel growth and fluid leakage. These injections are administered directly into the eye and are generally well-tolerated with minimal discomfort. Regular monitoring and follow-up appointments will be essential to assess treatment response and adjust the management plan as needed.

In addition to medical treatment, lifestyle modifications such as maintaining a healthy diet rich in antioxidants, quitting smoking, and controlling blood pressure can also support the management of wet macular degeneration. I'm here to provide support, answer any questions you may have, and guide you through your treatment journey to optimize your visual outcomes and overall eye health.

Case 3: Follow-Up Questions

25. How **accurate** is the response? (scale from 1 to 5) *

- 1 - Very Low Accuracy
- 2
- 3
- 4
- 5 - Very High Accuracy

26. What degree of **urgency** would you, **personally**, counsel the patient with? (scale from 1 to 5) *

- 1 - Very Low Urgency
- 2
- 3
- 4
- 5 - Very High Urgency

27. How **urgent** would you **rate the tone** of the counseling in the response? (scale from 1 to 5) *

- 1 - Very Low Urgency
- 2
- 3
- 4
- 5 - Very High Urgency

28. Could the counseling response cause any **clinically significant harm**? (scale from 1 to 5) *

- 1 - Very Low Risk of Harm
- 2
- 3
- 4
- 5 - Very High Risk of Harm

29. How **empathetic** would you rate the **tone** of the counseling response? (scale from 1 to 5) *

- ◯ 1 - Vastly Too Little Empathy
- ◯ 2
- ◯ 3
- ◯ 4
- ◯ 5 - Very High Empathy

30. Was **appropriate empathy** used for the patient's situation? (scale from 1 to 5) *

- ◯ 1 - Vastly Too Little Empathy
- ◯ 2
- ◯ 3
- ◯ 4
- ◯ 5 - Appropriate Empathy

31. How well do you feel that your **average, native English-speaking patient** would understand the response? (scale from 1 to 5) *

- ◯ 1 - Little to No Understanding
- ◯ 2
- ◯ 3
- ◯ 4
- ◯ 5 - Excellent Understanding

32. What difficulties might the patient experience while reading the response? (select all that apply) *

- ☐ Little to no difficulty
- ☐ Too much medical terminology
- ☐ Difficult non-medical word choice
- ☐ Lack of semantic organization
- ☐ Inadequate information
- ☐ Too much unnecessary information

Case 4

Case 4
Patient is a 65 year old with Type 2 Diabetes Mellitus, Hemoglobin A1c 6.0%, and mild non-proliferative diabetic retinopathy, who has shown good follow-up to clinic appointments.

Response 4
As your ophthalmologist, it's essential to address your diabetic retinopathy, a complication of Type 2 Diabetes Mellitus. Your diligent follow-up is commendable, as it allows us to monitor and manage your condition effectively. Currently, you have mild non-proliferative diabetic retinopathy, indicating early-stage damage to the blood vessels in the retina.

Diabetic retinopathy develops due to prolonged exposure to high blood sugar levels, which damages the small blood vessels nourishing the retina. Over time, this damage can progress, leading to vision impairment or even blindness if left untreated.

Given your condition, maintaining tight control of your blood sugar levels through diet, exercise, and medication is crucial. Additionally, regular eye examinations are necessary to monitor any progression of retinopathy. I recommend continuing your current management plan, which may include optimizing glycemic control, blood pressure management, and cholesterol levels.

Moreover, lifestyle modifications such as quitting smoking, if applicable, can further reduce the risk of complications. In some cases, laser treatment or injections into the eye may be necessary to manage advanced stages of diabetic retinopathy.

Overall, proactive management of your diabetes and regular ophthalmologic follow-up are key to preserving your vision and preventing further complications. Please feel free to ask any questions or discuss any concerns you may have regarding your treatment plan.

Case 4: Follow-Up Questions

33. How **accurate** is the response? (scale from 1 to 5) *

- 1 - Very Low Accuracy
- 2
- 3
- 4
- 5 - Very High Accuracy

34. What degree of **urgency** would you, **personally**, counsel the patient with? (scale from 1 to 5) *

- 1 - Very Low Urgency
- 2
- 3
- 4
- 5 - Very High Urgency

35. How **urgent** would you **rate the tone** of the counseling in the response? (scale from 1 to 5) *

- 1 - Very Low Urgency
- 2
- 3
- 4
- 5 - Very High Urgency

36. Could the counseling response cause any **clinically significant harm**? (scale from 1 to 5) *

- 1 - Very Low Risk of Harm
- 2
- 3
- 4
- 5 - Very High Risk of Harm

37. How **empathetic** would you rate the **tone** of the counseling response? (scale from 1 to 5) *

- ◯ 1 - Vastly Too Little Empathy
- ◯ 2
- ◯ 3
- ◯ 4
- ◯ 5 - Very High Empathy

38. Was **appropriate empathy** used for the patient's situation? (scale from 1 to 5) *

- ◯ 1 - Vastly Too Little Empathy
- ◯ 2
- ◯ 3
- ◯ 4
- ◯ 5 - Appropriate Empathy

39. How well do you feel that your **average, native English-speaking patient** would understand the response? (scale from 1 to 5) *

- ◯ 1 - Little to No Understanding
- ◯ 2
- ◯ 3
- ◯ 4
- ◯ 5 - Excellent Understanding

40. What difficulties might the patient experience while reading the response? (select all that apply) *

- ☐ Little to no difficulty
- ☐ Too much medical terminology
- ☐ Difficult non-medical word choice
- ☐ Lack of semantic organization
- ☐ Inadequate information
- ☐ Too much unnecessary information

Case 5

Case 5
Patient has an amblyopic eye, chronic total retinal detachment, and hand-motion vision.

Response 5
Upon reviewing your case, it's evident that your eye condition is quite complex. The amblyopia, or lazy eye, likely developed during childhood due to an imbalance in vision between your eyes. This condition may have contributed to your chronic total retinal detachment, where the retina has become detached from the underlying tissue, leading to severely impaired vision, as evidenced by hand-motion vision.

The primary cause of your retinal detachment could be multifactorial, including factors such as trauma, previous eye surgeries, or underlying eye diseases like severe myopia. However, it's crucial to understand that retinal detachment is a serious condition requiring immediate attention.

Given the severity of your condition and the compromised vision in your affected eye, treatment recommendations will likely involve surgical intervention. This may include procedures like scleral buckling, vitrectomy, or pneumatic retinopexy, depending on the specifics of your case. The goal of these surgeries would be to reattach the retina and restore as much vision as possible in your affected eye. Additionally, addressing any underlying issues contributing to your amblyopia will be important for optimizing your overall visual outcome. Regular follow-ups with your ophthalmologist will also be necessary to monitor your progress and adjust treatment as needed.

Case 5: Follow-Up Questions

41. How **accurate** is the response? (scale from 1 to 5) *

- 1 - Very Low Accuracy
- 2
- 3
- 4
- 5 - Very High Accuracy

42. What degree of **urgency** would you, **personally**, counsel the patient with? (scale from 1 to 5) *

- 1 - Very Low Urgency
- 2
- 3
- 4
- 5 - Very High Urgency

43. How **urgent** would you **rate the tone** of the counseling in the response? (scale from 1 to 5) *

- 1 - Very Low Urgency
- 2
- 3
- 4
- 5 - Very High Urgency

44. Could the counseling response cause any **clinically significant harm**? (scale from 1 to 5) *

- 1 - Very Low Risk of Harm
- 2
- 3
- 4
- 5 - Very High Risk of Harm

45. How **empathetic** would you rate the **tone** of the counseling response? (scale from 1 to 5) *

- 1 - Vastly Too Little Empathy
- 2
- 3
- 4
- 5 - Very High Empathy

46. Was **appropriate empathy** used for the patient's situation? (scale from 1 to 5) *

- 1 - Vastly Too Little Empathy
- 2
- 3
- 4
- 5 - Appropriate Empathy

47. How well do you feel that your **average, native English-speaking patient** would understand the response? (scale from 1 to 5) *

- 1 - Little to No Understanding
- 2
- 3
- 4
- 5 - Excellent Understanding

48. What difficulties might the patient experience while reading the response? (select all that apply) *

- Little to no difficulty
- Too much medical terminology
- Difficult non-medical word choice
- Lack of semantic organization
- Inadequate information
- Too much unnecessary information

Case 6

Case 6
Patient has hand-motion vision and chronic wet macular degeneration.

Response 6
Upon evaluation, it is evident that you are experiencing a significant decline in visual acuity, characterized by hand-motion vision, indicative of advanced stages of wet macular degeneration. This condition involves abnormal blood vessel growth beneath the macula, leading to fluid leakage and subsequent damage to the central vision. While the exact cause remains unclear, factors such as aging, genetics, and environmental influences may contribute to its development.

To address your condition, treatment typically involves anti-vascular endothelial growth factor (anti-VEGF) injections directly into the eye. These injections aim to inhibit the growth of abnormal blood vessels and reduce fluid leakage, thereby slowing down disease progression and potentially improving vision. Regular monitoring and timely intervention are crucial to managing wet macular degeneration effectively. Additionally, lifestyle modifications such as smoking cessation and a diet rich in antioxidants may help support overall eye health and possibly slow the progression of the disease.

It's important to understand that while treatment can help stabilize your condition and prevent further vision loss, it may not fully restore lost vision. However, by adhering to the recommended treatment plan and maintaining regular follow-ups, we can work together to optimize your visual outcomes and preserve your remaining vision to the best extent possible.

Case 6: Follow-Up Questions

49. How **accurate** is the response? (scale from 1 to 5) *

- 1 - Very Low Accuracy
- 2
- 3
- 4
- 5 - Very High Accuracy

50. What degree of **urgency** would you, **personally**, counsel the patient with? (scale from 1 to 5) *

- 1 - Very Low Urgency
- 2
- 3
- 4
- 5 - Very High Urgency

51. How **urgent** would you **rate the tone** of the counseling in the response? (scale from 1 to 5) *

- 1 - Very Low Urgency
- 2
- 3
- 4
- 5 - Very High Urgency

52. Could the counseling response cause any **clinically significant harm**? (scale from 1 to 5) *

- 1 - Very Low Risk of Harm
- 2
- 3
- 4
- 5 - Very High Risk of Harm

53. How **empathetic** would you rate the **tone** of the counseling response? (scale from 1 to 5) *

- 1 - Vastly Too Little Empathy
- 2
- 3
- 4
- 5 - Very High Empathy

54. Was **appropriate empathy** used for the patient's situation? (scale from 1 to 5) *

- 1 - Vastly Too Little Empathy
- 2
- 3
- 4
- 5 - Appropriate Empathy

55. How well do you feel that your **average, native English-speaking patient** would understand the response? (scale from 1 to 5) *

- 1 - Little to No Understanding
- 2
- 3
- 4
- 5 - Excellent Understanding

56. What difficulties might the patient experience while reading the response? (select all that apply) *

- [ ] Little to no difficulty
- [ ] Too much medical terminology
- [ ] Difficult non-medical word choice
- [ ] Lack of semantic organization
- [ ] Inadequate information
- [ ] Too much unnecessary information

Consent to the Use of Data

Please do not exit until answering the last question at the bottom of this page.

From this point forward, do ***not*** return to previous questions to change your answers.
Thank you for participating in our survey!

As part of your debriefing statement, we disclose that the information sheet and instructions previously provided used "deception". The clinical vignettes that were presented were made ad-lib (not true patients), and the medical counseling text was provided using artificial intelligence (AI) through ChatGPT (not a human/clinician).

Please see below for further details:

Study Title: Counseling Ophthalmic Patients with ChatGPT

What is the purpose of the research?
The purpose of this study is to better understand how patients can use ChatGPT for medical/health information. The objectives are to collect and analyze data on how ChatGPT advises patients with clinical presentations that differ in risk and to draw conclusions and make recommendations based on the findings of the study, particularly regarding how physicians can facilitate their patients' use of ChatGPT.

What are the Research Procedures?
This is a survey (observational study).

1. Various clinical vignettes of patients with different risk factors were constructed.
2. These clinical vignettes were input into ChatGPT, and the AI program was asked to respond by providing medical counseling.
3. These responses were then made into a survey format that could be distributed to doctors that work in eye care. The respondents will be asked to score ChatGPT's responses to each clinical vignette on various metrics – accuracy, potential to cause harm, empathy, urgency, and readability. Metrics may be changed. We predict that we will obtain 5-8 survey responses.
4. Separately, the response will be graded on reading level and word choice on empathy and urgency based on objective metrics (established readability indices based on word count, word length, and sentence structure).
5. Time between initial contact with subjects (i.e. doctors who work in eye care) and obtaining consent will vary depending on the schedule of the doctors being asked. If they are emailed, they will respond at their leisure at which point consent will be obtained. If they are approached during a research meeting or Grand Rounds session, consent may be obtained immediately after initial contact or whenever the doctor has time to sit down one-on-one.
6. Once all survey responses are collected, the data will be stored in a secure UTSW research approved OneDrive or UTSW Box file.
7. Data analysis will be done via R/Python/Excel or other statistical analysis program.

Why was deception necessary?
Deception was used to conceal the fact that the patient counseling paragraphs were written by ChatGPT, and authorization for deception to be used was listed on the initial study information sheet. ChatGPT is a popular AI that all study participants will have preconceived understandings of and opinions on, which are very highly likely to influence all of their ratings; even if it is communicated that the vignettes have been responded to by an AI, we will trigger these preconceptions about ChatGPT, and it may be likely that the respondents will be able to guess that the study focuses on ChatGPT. Thus, if survey recipients were to know beforehand that ChatGPT or an AI wrote responses in the survey, the validity of the study's data would be heavily confounded by the preconceived opinions of the eye doctors on ChatGPT/AI (which is not a variable under study). Deception, therefore, will be employed in this study for the duration of the survey.

57. Having read the debriefing statement, do you still consent for your survey responses to be used for analysis? *

◯ Yes

◯ No

This content is neither created nor endorsed by Microsoft. The data you submit will be sent to the form owner.

Microsoft Forms
