# Supplementary material for: Assessing retina-specific ophthalmic counseling generated by an early public large language model across different levels of clinical urgency
Source: Front Digit Health. 2026 Jul 1;8:1849883. doi: 10.3389/fdgth.2026.1849883 (PMC13368933; doi:10.3389/fdgth.2026.1849883)
Supplement: Supplementary file 4 [file Datasheet4.pdf]

Supplement 4. All variables as stratified by individuals' urgency score ratings

Variable	Rating of Vignette Urgency in Vignette Response					
	**1**<br>N = 5[1]	**2**<br>N = 14[1]	**3**<br>N = 28[1]	**4**<br>N = 33[1]	**5**<br>N = 28[1]	**p-value**[2]
Rating of response accuracy	5.00 (4.00, 5.00)	3.50 (2.00, 4.00)	4.00 (3.50, 5.00)	5.00 (4.00, 5.00)	5.00 (4.00, 5.00)	0.007
1	0 (0%)	0 (0%)	0 (0%)	0 (0%)	0 (0%)	
2	1 (20%)	4 (29%)	1 (3.6%)	2 (6.1%)	0 (0%)	
3	0 (0%)	3 (21%)	6 (21%)	1 (3.0%)	2 (7.1%)	
4	1 (20%)	4 (29%)	10 (36%)	13 (39%)	7 (25%)	
5	3 (60%)	3 (21%)	11 (39%)	17 (52%)	19 (68%)	
Rating of GPT response's urgency	2.00 (1.00, 3.00)	3.00 (2.00, 3.00)	3.00 (3.00, 3.50)	4.00 (3.00, 4.00)	4.00 (3.50, 4.00)	<0.001
1	2 (40%)	0 (0%)	0 (0%)	0 (0%)	0 (0%)	
2	1 (20%)	5 (36%)	1 (3.6%)	2 (6.1%)	1 (3.6%)	
3	1 (20%)	6 (43%)	20 (71%)	11 (33%)	6 (21%)	
4	1 (20%)	3 (21%)	7 (25%)	18 (55%)	16 (57%)	
5	0 (0%)	0 (0%)	0 (0%)	2 (6.1%)	5 (18%)	
Rating of clinically significant harm	1.00 (1.00, 1.00)	2.00 (2.00, 3.00)	1.00 (1.00, 2.50)	2.00 (1.00, 2.00)	2.00 (1.00, 2.00)	0.2
1	4 (80%)	3 (21%)	15 (54%)	14 (42%)	13 (46%)	
2	0 (0%)	5 (36%)	6 (21%)	14 (42%)	9 (32%)	
3	1 (20%)	5 (36%)	6 (21%)	4 (12%)	6 (21%)	
4	0 (0%)	1 (7.1%)	1 (3.6%)	1 (3.0%)	0 (0%)	
5	0 (0%)	0 (0%)	0 (0%)	0 (0%)		
Rating of response empathy	3.00 (2.00, 4.00)	3.00 (2.00, 3.00)	3.00 (2.00, 4.00)	3.00 (2.00, 4.00)	4.00 (3.00, 4.00)	0.12
1	0 (0%)	0 (0%)	2 (7.1%)	2 (6.1%)	0 (0%)	
2	2 (40%)	5 (36%)	6 (21%)	8 (24%)	5 (18%)	
3	1 (20%)	7 (50%)	10 (36%)	14 (42%)	6 (21%)	
4	2 (40%)	2 (14%)	7 (25%)	5 (15%)	14 (50%)	
5	0 (0%)	0 (0%)	3 (11%)	4 (12%)	3 (11%)	
Rating of empathy level appropriateness	3.00 (3.00, 4.00)	3.00 (3.00, 4.00)	3.00 (3.00, 5.00)	3.00 (3.00, 4.00)	4.00 (3.00, 5.00)	0.4
1	0 (0%)	1 (7.1%)	2 (7.1%)	2 (6.1%)	0 (0%)	
2	1 (20%)	2 (14%)	3 (11%)	6 (18%)	5 (18%)	
3	2 (40%)	7 (50%)	10 (36%)	10 (30%)	6 (21%)	
4	1 (20%)	3 (21%)	4 (14%)	8 (24%)	7 (25%)	
5	1 (20%)	1 (7.1%)	9 (32%)	7 (21%)	10 (36%)	
Rating of understandability to average, native	3.00 (2.00, 4.00)	3.00 (2.00, 4.00)	3.00 (2.00, 4.50)	3.00 (2.00, 4.00)	3.50 (3.00, 4.50)	0.5

English-speaking patient						
1	1 (20%)	1 (7.1%)	2 (7.1%)	2 (6.1%)	1 (3.6%)	
2	1 (20%)	5 (36%)	7 (25%)	7 (21%)	4 (14%)	
3	1 (20%)	4 (29%)	9 (32%)	13 (39%)	9 (32%)	
4	1 (20%)	3 (21%)	3 (11%)	5 (15%)	7 (25%)	
5	1 (20%)	1 (7.1%)	7 (25%)	6 (18%)	7 (25%)	
Response Difficulties: Little to no difficulties	1 (20%)	1 (7.1%)	8 (29%)	9 (27%)	7 (25%)	0.6
Response Difficulties: Too much medical terminology	3 (60%)	8 (57%)	15 (54%)	15 (45%)	12 (43%)	0.8
Response Difficulties: Difficult non-medical word choice	3 (60%)	7 (50%)	15 (54%)	14 (42%)	10 (36%)	0.7
Response Difficulties: Lack of semantic organization	0 (0%)	2 (14%)	4 (14%)	2 (6.1%)	2 (7.1%)	0.7
Response Difficulties: Inadequate information	0 (0%)	5 (36%)	5 (18%)	6 (18%)	4 (14%)	0.4
Response Difficulties: Too much unnecessary information	3 (60%)	7 (50%)	9 (32%)	11 (33%)	8 (29%)	0.5
Risk Level						<0.001
High Urgency	0 (0%)	0 (0%)	3 (11%)	24 (73%)	27 (96%)	
Low Urgency	5 (100%)	14 (100%)	25 (89%)	9 (27%)	1 (3.6%)	
Disease						0.002
Age-Related Macular Degeneration	1 (20%)	5 (36%)	7 (25%)	19 (58%)	4 (14%)	
Diabetic Retinopathy	3 (60%)	5 (36%)	13 (46%)	8 (24%)	7 (25%)	
Retinal Detachment	1 (20%)	4 (29%)	8 (29%)	6 (18%)	17 (61%)	

[1]n (%)

[2]Kruskal-Wallis rank sum test
