# Supplementary material for: Assessing retina-specific ophthalmic counseling generated by an early public large language model across different levels of clinical urgency
Source: Front Digit Health. 2026 Jul 1;8:1849883. doi: 10.3389/fdgth.2026.1849883 (PMC13368933; doi:10.3389/fdgth.2026.1849883)
Supplement: Supplementary file 5 [file Datasheet5.pdf]

Supplement 5. Descriptive statistics of survey responses by disease by risk level among retinal subspecialists

Characteristic	Age-Related Macular Degeneration			Diabetic Retinopathy			Retinal Detachment		
	High Urgency N = 11[1]	Low Urgency N = 11[1]	p-value[2]	High Urgency N = 11[1]	Low Urgency N = 11[1]	p-value[2]	High Urgency N = 11[1]	Low Urgency N = 11[1]	p-value[2]
**Rating of response accuracy**	5.00 (4.00, 5.00)	4.00 (3.00, 5.00)	0.3	5.00 (4.00, 5.00)	5.00 (4.00, 5.00)	0.3	5.00 (5.00, 5.00)	3.00 (3.00, 5.00)	0.005
**Rating of the vignette's urgency**	4.00 (4.00, 5.00)	3.00 (2.00, 4.00)	<0.001	5.00 (4.00, 5.00)	3.00 (1.00, 3.00)	<0.001	5.00 (5.00, 5.00)	3.00 (2.00, 4.00)	<0.001
**Rating of GPT response's urgency**	4.00 (3.00, 4.00)	3.00 (2.00, 4.00)	0.13	4.00 (4.00, 5.00)	3.00 (2.00, 3.00)	<0.001	4.00 (4.00, 4.00)	3.00 (3.00, 4.00)	0.068
**Rating of clinically significant harm**	1.00 (1.00, 2.00)	1.00 (1.00, 2.00)	0.7	1.00 (1.00, 2.00)	1.00 (1.00, 2.00)	0.8	1.00 (1.00, 3.00)	2.00 (1.00, 3.00)	0.6
**Rating of response empathy**	4.00 (3.00, 4.00)	3.00 (2.00, 3.00)	0.007	3.00 (2.00, 4.00)	3.00 (3.00, 4.00)	0.3	4.00 (2.00, 4.00)	2.00 (2.00, 3.00)	0.052
**Rating of empathy level appropriateness**	4.00 (3.00, 5.00)	3.00 (3.00, 3.00)	0.014	4.00 (2.00, 4.00)	4.00 (3.00, 5.00)	0.3	4.00 (2.00, 5.00)	3.00 (2.00, 4.00)	0.2
**Rating of understandability to average, native English-speaking patient**	4.00 (3.00, 5.00)	3.00 (2.00, 3.00)	0.030	3.00 (2.00, 4.00)	4.00 (3.00, 4.00)	0.3	4.00 (3.00, 4.00)	3.00 (2.00, 3.00)	0.042
**Response Difficulties: Little to no difficulties**	3 (27%)	1 (9.1%)	0.6	3 (27%)	2 (18%)	>0.9	1 (9.1%)	0 (0%)	>0.9
**Response Difficulties: Too much medical terminology**	3 (27%)	8 (73%)	0.033	5 (45%)	5 (45%)	>0.9	6 (55%)	6 (55%)	>0.9
**Response Difficulties: Difficult non-medical word choice**	4 (36%)	7 (64%)	0.2	7 (64%)	5 (45%)	0.4	4 (36%)	6 (55%)	0.4

**Response Difficulties: Lack of semantic organization**	0 (0%)	2 (18%)	0.5	0 (0%)	0 (0%)	N/A	1 (9.1%)	2 (18%)	>0.9
**Response Difficulties: Inadequate information**	3 (27%)	5 (45%)	0.7	0 (0%)	1 (9.1%)	>0.9	4 (36%)	5 (45%)	>0.9
**Response Difficulties: Too much unnecessary information**	3 (27%)	2 (18%)	>0.9	4 (36%)	5 (45%)	>0.9	3 (27%)	5 (45%)	0.7

[1]Median (Q1, Q3); n (%)

[2]Kruskal-Wallis rank sum test; Fisher's exact test; Pearson's Chi-squared test
