# Supplementary material for: Assessing retina-specific ophthalmic counseling generated by an early public large language model across different levels of clinical urgency
Source: Front Digit Health. 2026 Jul 1;8:1849883. doi: 10.3389/fdgth.2026.1849883 (PMC13368933; doi:10.3389/fdgth.2026.1849883)
Supplement: Supplementary file 6 [file Datasheet6.pdf]

Supplement 6. Descriptive statistics of survey responses by urgency score among retinal subspecialists only

Characteristic	Urgency Score (Median Physician Rating of Vignette's Urgency)			
	**3**<br>N = 33[1]	**4**<br>N = 11[1]	**5**<br>N = 22[1]	**p-value**[2]
Rating of response accuracy	4.00 (3.00, 5.00)	5.00 (4.00, 5.00)	5.00 (5.00, 5.00)	0.007
1	0 (0%)	0 (0%)	0 (0%)	
2	4 (12%)	0 (0%)	0 (0%)	
3	6 (18%)	1 (9.1%)	0 (0%)	
4	10 (30%)	4 (36%)	5 (23%)	
5	13 (39%)	6 (55%)	17 (77%)	
Rating of GPT response's urgency	3.00 (3.00, 3.00)	4.00 (3.00, 4.00)	4.00 (4.00, 4.00)	<0.001
1	2 (6.1%)	0 (0%)	0 (0%)	
2	5 (15%)	1 (9.1%)	0 (0%)	
3	18 (55%)	4 (36%)	3 (14%)	
4	7 (21%)	5 (45%)	15 (68%)	
5	1 (3.0%)	1 (9.1%)	4 (18%)	
Rating of clinically significant harm	1.00 (1.00, 2.00)	1.00 (1.00, 2.00)	1.00 (1.00, 2.00)	0.8
1	17 (52%)	6 (55%)	12 (55%)	
2	9 (27%)	5 (45%)	5 (23%)	
3	5 (15%)	0 (0%)	5 (23%)	
4	2 (6.1%)	0 (0%)	0 (0%)	
5	0 (0%)	0 (0%)	0 (0%)	
Rating of response empathy	3.00 (2.00, 3.00)	4.00 (3.00, 4.00)	3.00 (2.00, 4.00)	0.037
1	0 (0%)	0 (0%)	1 (4.5%)	
2	12 (36%)	0 (0%)	5 (23%)	
3	14 (42%)	5 (45%)	7 (32%)	
4	6 (18%)	5 (45%)	8 (36%)	
5	1 (3.0%)	1 (9.1%)	1 (4.5%)	

Rating of empathy level appropriateness	3.00 (3.00, 4.00)	4.00 (3.00, 5.00)	4.00 (2.00, 5.00)	0.2
1	1 (3.0%)	0 (0%)	1 (4.5%)	
2	4 (12%)	0 (0%)	5 (23%)	
3	17 (52%)	3 (27%)	4 (18%)	
4	4 (12%)	4 (36%)	6 (27%)	
5	7 (21%)	4 (36%)	6 (27%)	
Rating of understandability to average, native English-speaking patient	3.00 (2.00, 4.00)	4.00 (3.00, 5.00)	3.00 (2.00, 4.00)	0.10
1	1 (3.0%)	0 (0%)	0 (0%)	
2	9 (27%)	0 (0%)	6 (27%)	
3	14 (42%)	5 (45%)	6 (27%)	
4	5 (15%)	3 (27%)	6 (27%)	
5	4 (12%)	3 (27%)	4 (18%)	
Response Difficulties: Little to no difficulties	3 (9.1%)	3 (27%)	4 (18%)	0.3
Response Difficulties: Too much medical terminology	19 (58%)	3 (27%)	11 (50%)	0.2
Response Difficulties: Difficult non-medical word choice	18 (55%)	4 (36%)	11 (50%)	0.6
Response Difficulties: Lack of semantic organization	4 (12%)	0 (0%)	1 (4.5%)	0.6
Response Difficulties: Inadequate information	11 (33%)	3 (27%)	4 (18%)	0.5
Response Difficulties: Too much unnecessary information	12 (36%)	3 (27%)	7 (32%)	0.9
**Risk Level**				**<0.001**
High Urgency	0 (0%)	11 (100%)	22 (100%)	
Low Urgency	33 (100%)	0 (0%)	0 (0%)	

[1]Median (Q1, Q3); n (%)

[2]Kruskal-Wallis rank sum test; Fisher's exact test; Pearson's Chi-squared test
