# Supplementary material for: Assessing retina-specific ophthalmic counseling generated by an early public large language model across different levels of clinical urgency
Source: Front Digit Health. 2026 Jul 1;8:1849883. doi: 10.3389/fdgth.2026.1849883 (PMC13368933; doi:10.3389/fdgth.2026.1849883)
Supplement: Supplementary file 7 [file Datasheet7.pdf]

Supplement 7. Survey Reliability Metrics

**Survey Question**	**Survey Reliability Statistic**
Rating of response accuracy[1]	0.87, 95% CI [0.658, 0.98]
Rating of the vignette's urgency[1]	0.97, 95% CI [0.92, 1.00]
Rating of GPT response's urgency[1]	0.88, 95% CI [0.662, 0.98]
Rating of clinically significant harm[1]	0.582, 95% CI [-0.1364, 0.93]
Rating of response empathy[1]	0.819, 95% CI [0.5080, 0.97]
Rating of empathy level appropriateness[1]	0.780, 95% CI [0.4029, 0.96]
Rating of understandability to average, native English-speaking patient[1]	0.77, 95% CI [0.364, 0.96]
Overall survey including all Likert-scale questions	0.94, 95% CI [0.91, 0.96]
Response Difficulties: various readability (Yes/No) questions[2]	0.103, p = 1.73e-14

[1]Intraclass correlation coefficient (ICC) using a two-way mixed effects model with average measures for consistency
[2]Fleiss' Kappa
